# Multimodal radiomics model with triple -timepoint contrast-enhanced ultrasound for precise diagnosis of C-TIRADS 4 thyroid nodules

**DOI:** 10.3389/fendo.2025.1639017

**Published:** 2025-08-19

**Authors:** Linlin Shao, Lili Zhang, Lifang Liu, Fangfang Sun, Hongyu Li, Tongfeng Liu, Feng Hu, Lirong Zhao

**Affiliations:** Ultrasound Diagnostic Center, The First Hospital of Jilin University, Changchun, China

**Keywords:** thyroid nodules, contrast-enhanced ultrasound, radiomics features, machine learning, C-TIRADS

## Abstract

**Objective:**

This study aims to construct a multimodal radiomics model based on contrast-enhanced ultrasound (CEUS) radiomic features, combined with conventional ultrasonography (US) images and clinical data, to evaluate its diagnostic efficacy in differentiating benign and malignant thyroid nodules (TNs) classified as C-TIRADS 4, and to assess the clinical application value of the model.

**Methods:**

This retrospective study enrolled 135 patients with C-TIRADS 4 thyroid nodules who underwent concurrent US and CEUS before FNA/surgery. From each case, one US image and three CEUS key frames (2s post-perfusion, peak enhancement, 2s post-peak) were selected. Patients were randomly split into training (n=108) and test (n=27) cohorts (8:2 ratio). ROIs were manually delineated (3D-Slicer), with radiomics features extracted (PyRadiomics) and selected via mRMR and LASSO. Six CEUS radiomics-based machine learning models (KNN, SVM, RF, XGBoost, LightGBM, SGD) were developed and evaluated using AUC, accuracy, sensitivity, specificity, and F1-score. The optimal classifier was used to build US-only, US+CEUS, and clinical+US+CEUS models. Statistical comparisons employed DeLong tests, calibration curves, and DCA.

**Results:**

The CEUS radiomics model demonstrated favorable diagnostic performance in differentiating benign and malignant C-TIRADS 4 thyroid nodules, with sensitivity, specificity, and accuracy of 0.875, 0.769, and 0.833, respectively. When CEUS radiomic features were combined with US features, the diagnostic performance of the CEUS radiomics model was comparable to that of the US+CEUS radiomics model (AUC: 0.813 vs. 0.829, P=0.005). Furthermore, the multimodal radiomics model integrating clinical data (clinical+US+CEUS radiomics model) achieved significantly improved diagnostic efficacy, with an AUC of 0.967, along with accuracy, sensitivity, specificity, and F1-score values of 0.815, 0.823, 0.792, and 0.884, respectively.

**Conclusion:**

Our study developed a high-performance multimodal diagnostic model through the innovative integration of radiomic features from three critical CEUS timepoints combined with conventional ultrasound and clinical data, establishing a novel decision-support tool for accurate noninvasive classification of C-TIRADS 4 thyroid nodules. The model’s superior diagnostic performance (AUC 0.967) demonstrates the transformative potential of multimodal integration in overcoming single-modality limitations and enhancing clinical decision-making, positioning this approach as a promising solution to mitigate unnecessary diagnostic procedures and overtreatment.

## 1 Introduction

In recent years, the rapid advancement of ultrasound imaging technology and its significantly improved diagnostic sensitivity, coupled with multiple factors such as dietary patterns and environmental influences, have led to a marked increase in both the incidence and detection rates of thyroid nodules (TNs). Conventional ultrasonography (US), as the primary auxiliary examination method for TNs, has been widely adopted in clinical practice. Statistics indicate that the prevalence rate of TNs exceeding 5 mm in maximum diameter detected by ultrasound reach 20.43% among the adult population in China (1). However, the majority of TNs are benign lesions, and even some malignant nodules often exhibit indolent growth patterns (2), suggesting that not all TNs require fine-needle aspiration (FNA).To standardize the diagnosis and management of TNs, numerous national and international thyroid associations have established ultrasound-based diagnostic classification systems (3–5), providing crucial guidance for differentiating benign and malignant TNs. Among these, the Chinese Guidelines for Ultrasound Malignancy Risk Stratification of Thyroid Nodules (C-TIRADS) (4) developed by the Superficial Organ and Vascular Study Group of the Chinese Medical Association Ultrasound Medicine Branch, holds significant clinical relevance. According to the C-TIRADS classification criteria, the malignancy risk for C-TIRADS category 3 nodules is less than 2%, while for C-TIRADS category 4 nodules, it ranges between 2% and 90%, and for C-TIRADS category 5 nodules, it exceeds 90%.Notably, the malignancy risk spectrum of C-TIRADS category 4 nodules is particularly broad, leading to unnecessary invasive procedures such as FNA or even surgical resection for certain benign nodules classified as C-TIRADS 4a or 4b (6). Therefore, developing a reliable and noninvasive diagnostic approach to more accurately distinguish benign from malignant C-TIRADS category 4 nodules—particularly those for which FNA is not recommended—holds substantial clinical significance and practical value.

Currently, contrast-enhanced ultrasound (CEUS) has been widely applied in the diagnosis of TNs (7). Utilizing microbubble contrast agents as pure blood pool imaging tracers, CEUS enables qualitative and quantitative analysis of macro- and microvascular morphology in TNs and surrounding tissues by observing dynamic perfusion patterns after intravenous injection and systemic circulation (8). Although standardized guidelines for CEUS in TN diagnosis remain lacking, it has become an important auxiliary tool for sonographers in clinical practice. Previous studies have demonstrated that combining conventional ultrasound with CEUS significantly improves diagnostic accuracy, sensitivity, and specificity for TNs (9–11). However, the diagnostic efficacy of CEUS in clinical practice is highly operator-dependent, with results often influenced by the subjective experience of sonographers. This subjectivity may lead to inter-observer variability, ultimately affecting the accuracy and reliability of clinical decision-making. Therefore, developing a more objective and quantitative analytical approach has become a key research focus.

Radiomics, as a prominent research area in medical imaging, has shown great potential in precision medicine. By extracting high-throughput quantitative features from medical images to construct diagnostic or predictive models, radiomics can effectively reduce subjective variability and experience-dependency among different physicians, providing a more objective reflection of imaging information and offering a promising tool for noninvasive precision diagnosis and treatment (12). Currently, ultrasound-based radiomics has demonstrated high diagnostic performance in various fields, including liver (13), breast (14)and thyroid (15)studies, providing novel technical approaches for the precise diagnosis of TNs. However, most previous studies have focused on building radiomics models based on two-dimensional ultrasound images (15, 16), Although some studies have utilized CEUS to differentiate TN characteristics (17, 18), they typically extracted features from a single peak-intensity frame of CEUS videos, which may inadequately capture dynamic blood flow changes in nodules. Only one study analyzed five key CEUS time points but did not incorporate multimodal data for comprehensive evaluation (19). To overcome these limitations, our study employs a multi-timepoint and multimodal fusion approach. Specifically, we selected three key CEUS frames—”the second second after perfusion initiation,” “peak enhancement time,” and “the second second after peak enhancement”—to construct a CEUS radiomics model, thereby comprehensively characterizing nodule hemodynamics and avoiding the constraints of single-timepoint analysis. Furthermore, we integrated conventional ultrasound features and clinical data to develop a multimodal radiomics model, evaluating the diagnostic performance differences among various models for C-TIRADS category 4 TNs. This approach aims to provide a more comprehensive solution for the precise diagnosis of TNs.

## 2 Materials and methods

### 2.1 Patients

**Figure 1:**
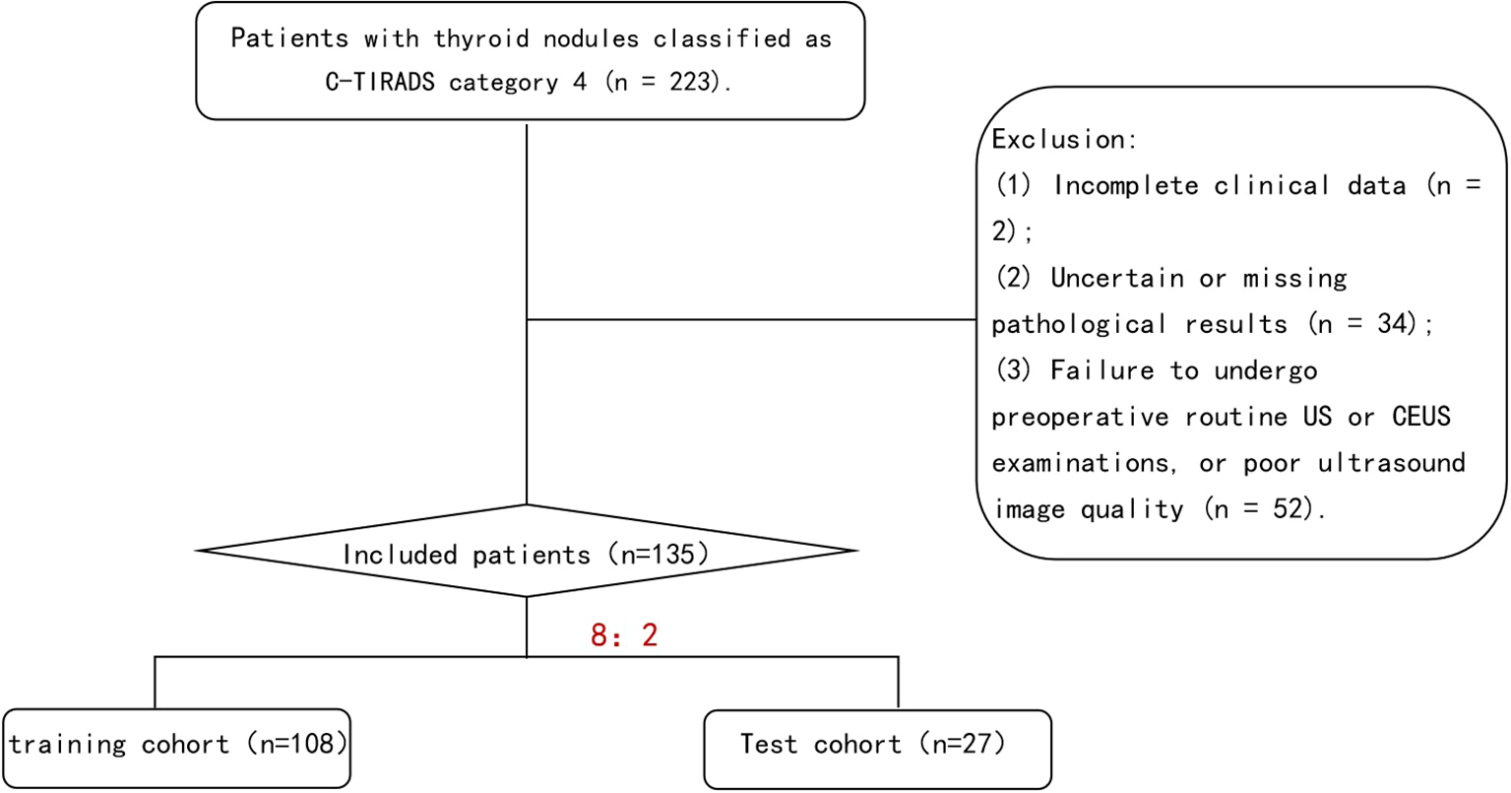
Flowchart of study population screening.

We retrospectively analyzed 135 patients with C-TIRADS category 4 TNs who underwent both US and CEUS examinations prior to fine-needle aspiration (FNA) or surgical resection at our institution between January 2021 and September 2024.Using histopathological results as the gold standard, the patients were stratified into benign and malignant groups. Through random sampling, the entire cohort was divided into a training cohort (n=108) and a test cohort (n=27) at an 8:2 ratio. The training cohort was utilized for model development, while the test cohort served for model validation (**Figure 1**). Inclusion Criteria: (1)Thyroid nodules classified as C-TIRADS category 4; (2)No history of thyroid surgery or FNA; (3)Completion of both conventional US and CEUS examinations with optimal image quality prior to intervention; (4)Availability of definitive pathological results from either FNA or surgical resection; (5)Absence of allergic reactions to the contrast agent SonoVue. Exclusion Criteria: (1) Incomplete clinical records;(2) Indeterminate or unavailable pathological results; (3) Failure to undergo pre-operative conventional US and CEUS examinations or suboptimal ultrasound image quality; (4) History of severe allergies or documented hypersensitivity to SonoVue contrast agent; (5) Pregnancy or lactation status. The clinical and ultrasonographic characteristics were well-balanced between the training (n=108) and test (n=27) cohorts (**Supplementary Table 1**). All compared parameters showed no statistically significant differences (P>0.05 for all), meeting the data requirements for model development.

### 2.2 Ultrasound examination protocol and image acquisition

The ultrasound examinations were performed using GE LOGIQ E9 and GE LOGIQ E11 color ultrasound diagnostic systems.

**Figure 2:**
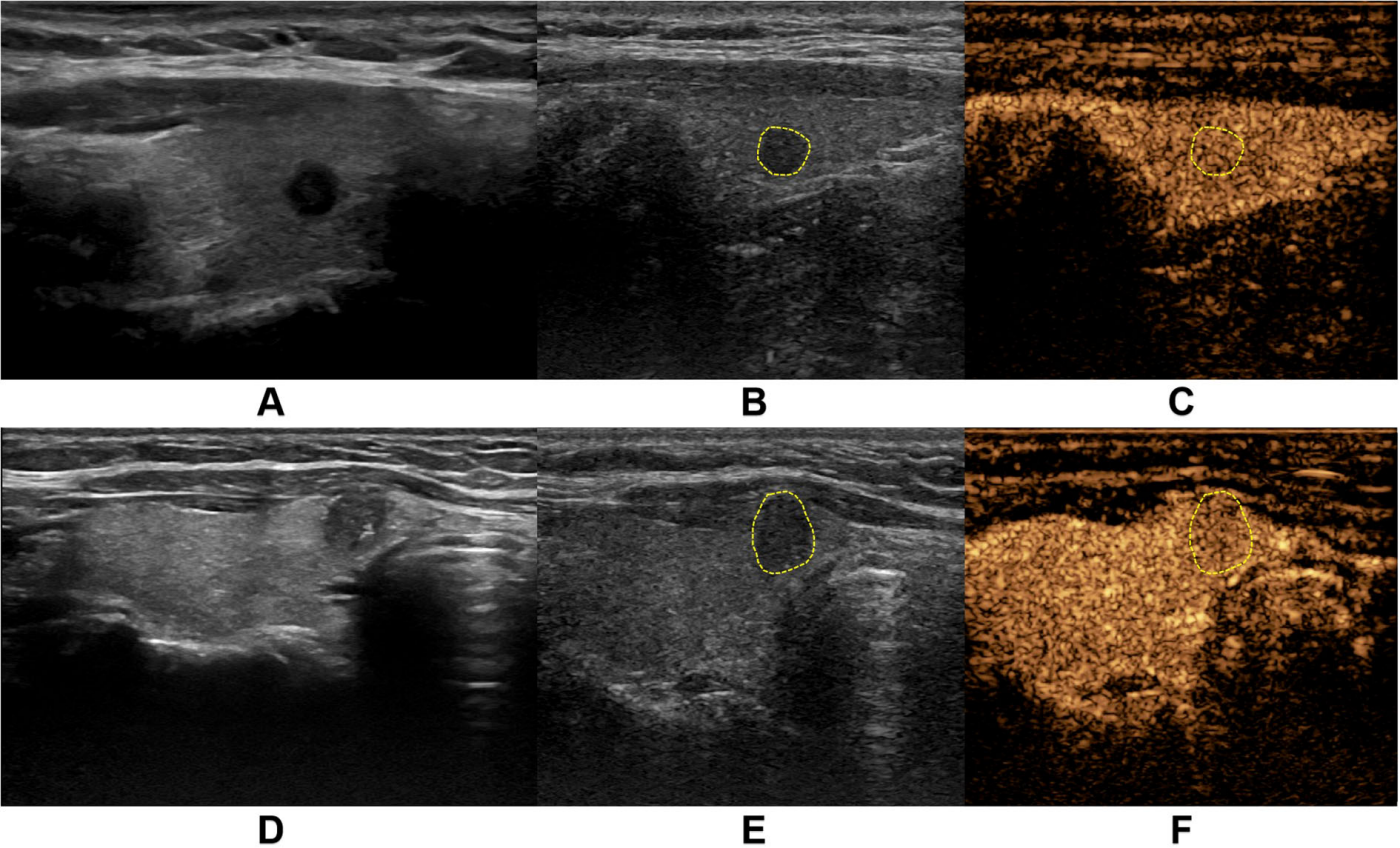
Representative ultrasound images from two thyroid nodule cases. **(A-C)** demonstrate a case from a middle-aged female patient with a markedly hypoechoic nodule (5.0 mm × 4.0 mm) in the right thyroid lobe. **(A)** shows the conventional grayscale ultrasound (US) image, while **(B, C)** display the corresponding two-dimensional and contrast-enhanced images at peak enhancement during contrast-enhanced ultrasound (CEUS), revealing isoenhancement pattern. Pathological diagnosis confirmed nodular goiter. **(D-F)** illustrate another middle-aged female patient with a solid hypoechoic nodule (6.1 mm × 7.8 mm) in the right thyroid lobe near the isthmus. **(D)** presents the conventional US image, with **(E, F)** showing the CEUS images at peak enhancement demonstrating hypoenhancement pattern. Final pathology confirmed papillary thyroid carcinoma.

US Image Acquisition: Patients were positioned in the supine position with adequate neck exposure while maintaining quiet breathing to minimize respiratory motion artifacts. Standardized scanning was performed by sonographers with over 5 years of thyroid ultrasound experience. Following C-TIRADS guidelines, the following nodule characteristics were evaluated and recorded: Maximum diameter and three-dimensional measurements; Internal echogenicity (marked hypoechoic, hypoechoic, isoechoic, or hyperechoic); Taller-than-wide shape (height/width ratio >1 or <1); Margins (ill-defined or smooth); Microcalcifications (present or absent). Imaging parameters (gain, depth, focus) were optimized for each examination. The largest cross-sectional grayscale image was saved for subsequent analysis (**Figure 2**).

CEUS Image Acquisition: The maximum diameter section of the target nodule was selected as the observation plane, with inclusion of surrounding normal thyroid tissue as reference. SonoVue contrast agent (Bracco SpA Inc, Milan, Italy) was prepared by injecting 5 mL of 0.9% sodium chloride solution into the vial, followed by vigorous shaking to form a homogeneous suspension. A 2.4 mL bolus of the contrast suspension was rapidly injected via the antecubital vein, immediately followed by a 5 mL saline flush. Timing commenced upon contrast injection, with maintenance of stable probe positioning throughout the examination. Patients were instructed to maintain quiet breathing and refrain from speaking, swallowing, or coughing. The entire contrast perfusion process was continuously monitored and recorded as dynamic cine loops. All examinations were performed by two sonologists with over 10 years of thyroid ultrasound experience, adhering strictly to standardized protocols to ensure consistent image quality and reproducible diagnostic results (**Figure 2**).

### 2.3 Region of interest segmentation on ultrasound images

**Figure 3:**
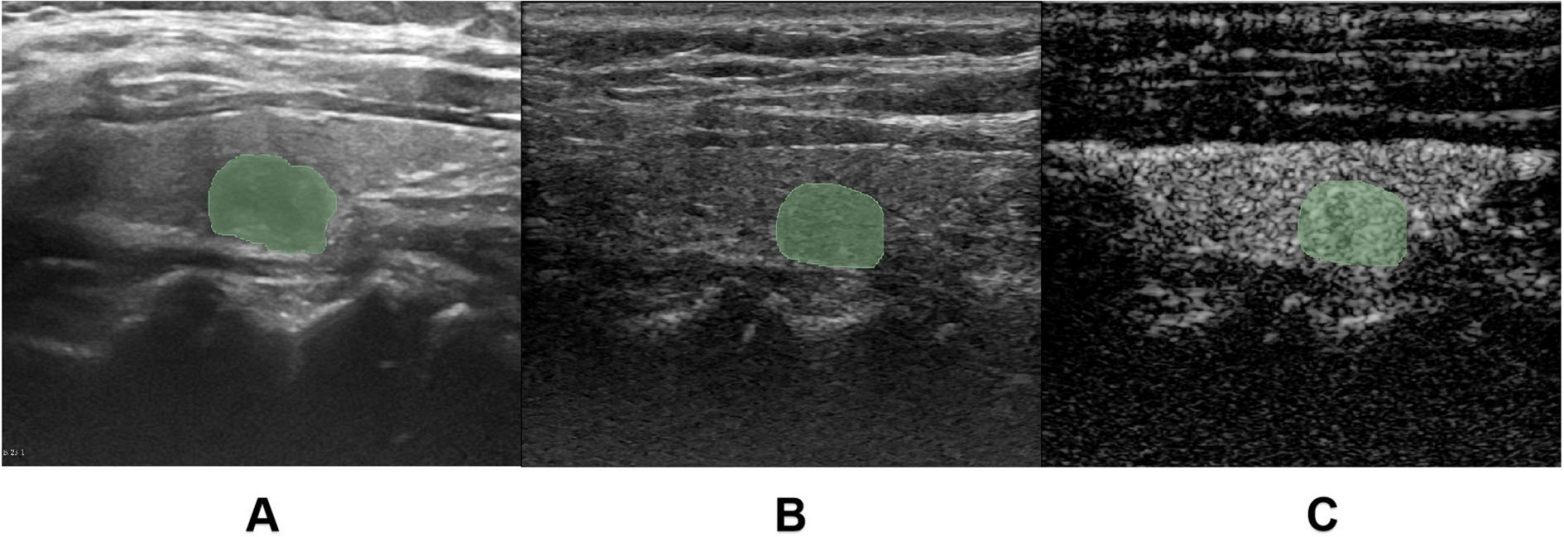
Region of interest (ROI) delineation. **(A)** demonstrates the manual ROI tracing on conventional grayscale ultrasound (US) images. **(B, C)** illustrate the ROI delineation process for contrast-enhanced ultrasound (CEUS) images, where the ROI was initially drawn on the grayscale reference image **(C)** corresponding to the key CEUS frame **(B)**, and subsequently mapped onto the contrast-enhanced image.

The CEUS dynamic cine loops were reviewed on the ultrasound system to identify three key timepoints: “2 seconds after contrast perfusion initiation”, “peak enhancement time” and “2 seconds post-peak enhancement”. For each target nodule, one conventional grayscale US image and six key-frame images (comprising both grayscale US and CEUS images at each timepoint) were exported (**Figure 3**). All images were imported into the medical image processing software 3D-Slicer (Version 5.6.2) for manual ROI delineation. The nodule ROI was manually traced directly on conventional US images to ensure complete coverage of the entire nodule area. To address potential boundary indistinctness of malignant nodules on CEUS images, we implemented a dual-modality segmentation protocol: First, ROIs were delineated on grayscale US images corresponding to each key timepoint to generate reference masks. These masks were then precisely mapped onto their paired CEUS images using the software’s registration tools, maintaining spatial consistency and measurement accuracy across imaging modalities (**Figure 3**).

### 2.4 Feature extraction and selection

**Figure 4:**
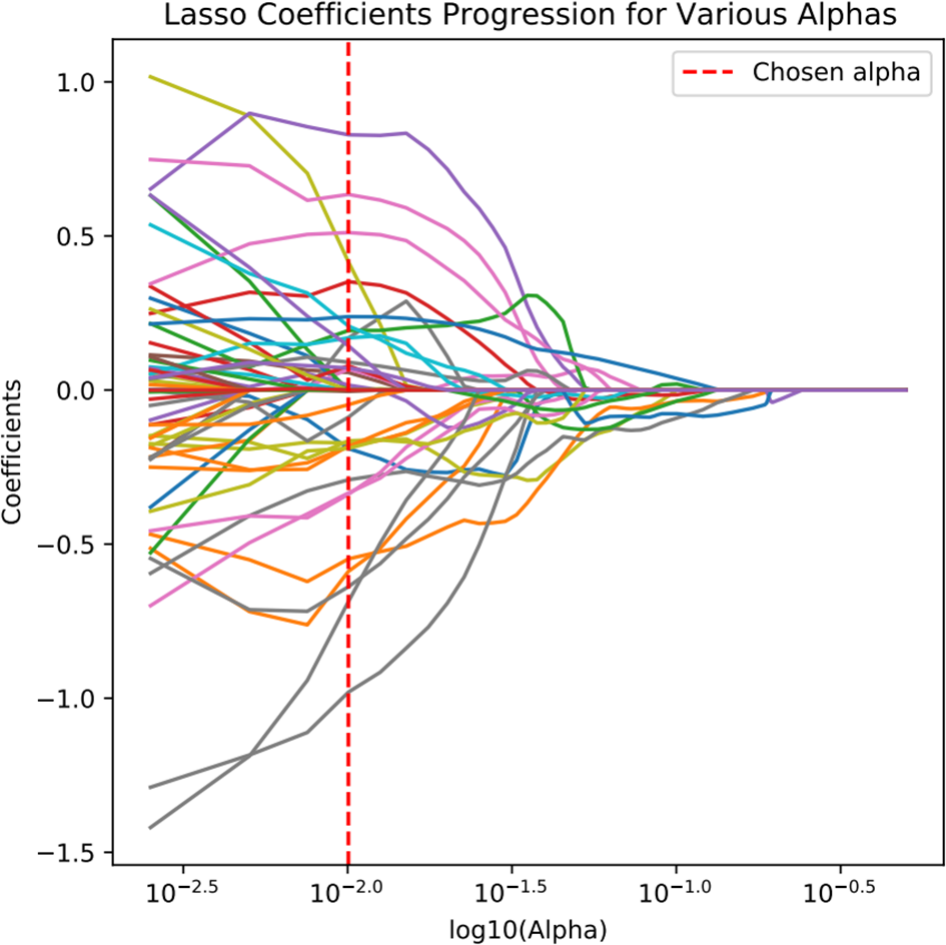
Feature selection using LASSO regression algorithm.

**Figure 5:**
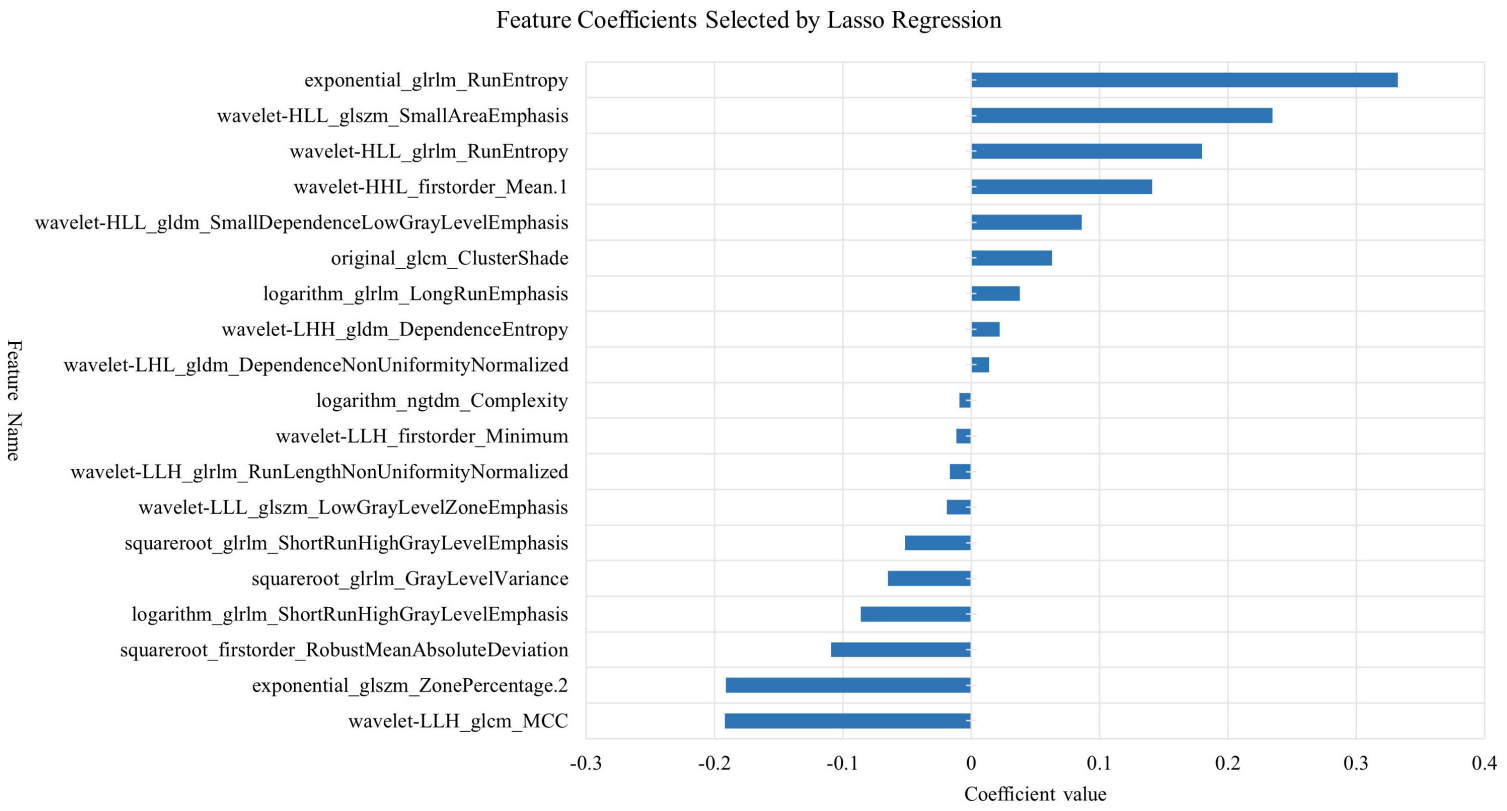
Feature Coefficient distribution selected by LASSO regression.

Radiomic feature extraction was performed using the open-source Python package PyRadiomics. The extracted features primarily consisted of three categories: first-order features describing fundamental statistical properties of voxel intensity distributions (including mean, median, standard deviation, skewness, and kurtosis); shape features encompassing both 2D and 3D morphological descriptors (such as nodule volume, surface area, and sphericity); and texture features quantifying tumor heterogeneity through various matrices including Gray Level Co-occurrence Matrix (GLCM), Gray Level Size Zone Matrix (GLSZM), Gray Level Run Length Matrix (GLRLM), Neighboring Gray Tone Difference Matrix (NGTDM), and Gray Level Dependence Matrix (GLDM), along with higher-order features derived from wavelet transformations. A total of 1,354 radiomic features were extracted from each image. To mitigate potential data bias and overfitting risks, all extracted radiomic features underwent standardized preprocessing using Z-score normalization. The feature selection process employed a two-step dimensionality reduction strategy: initially applying the Maximum Relevance Minimum Redundancy (mRMR) algorithm to training cohort data to identify feature subsets exhibiting high correlation with target variables while maintaining low inter-feature redundancy, followed by further refinement using Least Absolute Shrinkage and Selection Operator (LASSO) regression to ultimately derive a radiomic feature cohort with significant diagnostic value (**Figures 4**, **5**).

### 2.5 Model development

**Figure 6:**
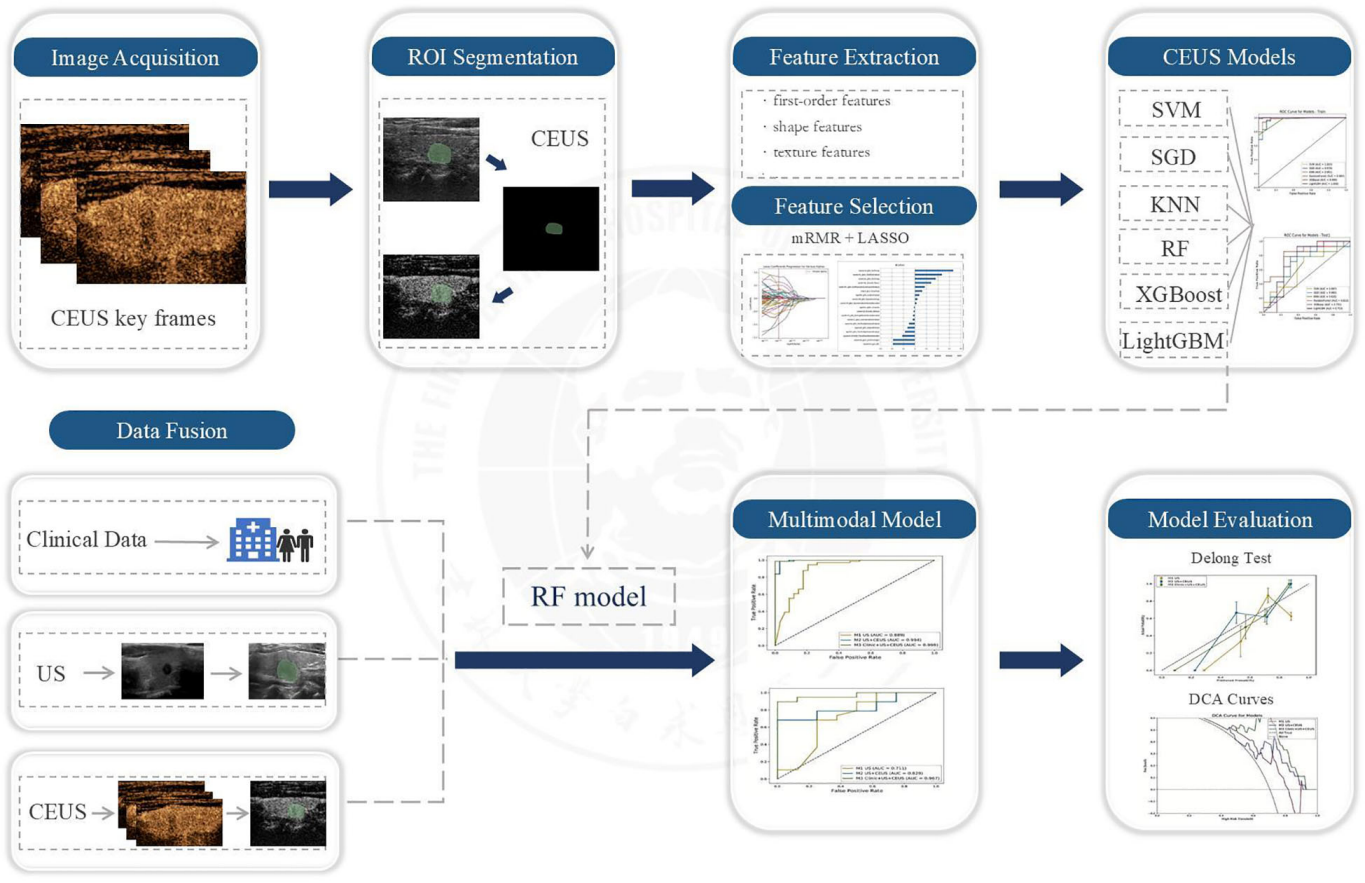
Schematic workflow diagram.

Based on the optimal radiomic features selected through dimensionality reduction, we initially constructed six machine learning models including Support Vector Machine (SVM), Stochastic Gradient Descent (SGD), K Nearest Neighbor (KNN), Random Forest (RF), Extreme Gradient Boosting (XGBoost), and Light Gradient Boosting Machine (LightGBM). To ensure optimal model performance and avoid overfitting or underfitting, we employed ten-fold cross-validation for parameter evaluation. The diagnostic performance of these six CEUS-based radiomic models was comprehensively assessed using multiple metrics including Receiver Operating Characteristic (ROC) curves, Area Under the Curve (AUC), accuracy, sensitivity, and specificity, through which the model demonstrating superior diagnostic efficacy was identified. Subsequently, this optimal model was applied to construct three additional radiomic models for further evaluation. The workflow scheme is illustrated in **Figure 6**.

### 2.6 Statistical analysis

Statistical analysis was performed using SPSS 27.0 software. Measurement data were expressed as mean ± standard deviation, and enumeration data were presented as frequency and percentage. Independent samples t-test was used for comparison of measurement data between groups, while chi-square test was employed for comparison of enumeration data between groups. The diagnostic performance of each model for TN characterization was comprehensively evaluated by calculating multiple indicators including AUC, accuracy, sensitivity, specificity and F1-score. DeLong test was used to compare AUC values among different machine learning models. Calibration curve and decision curve analysis (DCA) were applied to assess the clinical utility of the models. All model training and evaluation were conducted on independent training and test sets to ensure the reliability and generalizability of evaluation results. A P value <0.05 was considered statistically significant.

## 3 Results

### 3.1 Comparison of clinical and sonographic characteristics

This retrospective study included 135 patients with C-TIRADS category 4 TNs, comprising 32 males and 103 females, with a mean age of 49.63 ± 10.47 years. Among the study samples, there were 42 benign nodules and 93 malignant nodules. The benign nodules primarily consisted of nodular goiter, adenoma, adenomatous nodular goiter, and inflammatory lesions. Among the malignant nodules, 92 were papillary thyroid carcinomas, and 1 was medullary carcinoma. The comparison of clinical and sonographic characteristics between the benign and malignant groups is presented in **Table 1**.

**Table 1:** Comparison of clinical and sonographic characteristics.

Characteristics	Benign (n=42)	Malignant (n=93)	*P-*value
Age (years)	52.55 ± 10.83	48.31 ± 10.09	0.029*
Sex			
Male	9 (21.4%)	23 (24.7%)	
Female	33 (78.6%)	70 (75.3%)	0.676
Maximum nodule diameter (mm)	12.42 ± 12.16	9.25 ± 5.64	0.040*
Location			
Isthmus	1 (2.4%)	6 (6.5%)	
Right lobe	22 (52.4%)	52 (55.9%)	
Left lobe	19 (45.2%)	35 (37.6%)	0.494
Echogenicity			
Markedly hypoechoic	0 (0.0%)	8 (8.6%)	
Hypoechoic	33 (78.6%)	83 (89.2%)	
Isoechoic	1 (2.4%)	0 (0.0%)	
Hyperechoic	1 (2.4%)	1 (1.1%)	
Mixed echogenicity	7 (16.7%)	1 (1.1%)	0.001*
Shape (Aspect ratio)			
<1	30 (71.4%)	41 (44.1%)	
>1	12 (28.6%)	52 (55.9%)	0.003*
Margin			
Regular	25 (59.5%)	36 (38.7%)	
Irregular	17 (40.5%)	57 (61.3%)	0.024*
Microcalcification			
Yes	11 (26.2%)	43 (46.2%)	
No	31 (73.8%)	50 (53.8%)	0.028*
Vascularity			
Yes	18 (42.9%)	52 (55.9%)	
No	24 (57.1%)	41 (44.1%)	0.160
Enhancement Intensity			
Hypoenhancement	13 (31.0%)	72 (77.4%)	
Isoenhancement	25 (59.5%)	20 (21.5%)	
Hyperenhancement	4 (9.5%)	1 (1.1%)	<0.001*
Enhancement homogeneity			
Homogeneous	17 (40.5%)	21 (22.6%)	
heterogeneous	25 (59.5%)	72 (77.4%)	0.032*
Enhancement directionality			
Centripetal	19 (45.2%)	68 (73.1%)	
Non-centripetal	23 (54.8%)	25 (26.9%)	0.002*

*P < 0.05 was considered to indicate a statistically significant difference.

The results (**Table 1**) demonstrated a statistically significant difference in age between the benign and malignant groups (P < 0.05), while no significant difference was observed in gender distribution (P > 0.05). Regarding sonographic characteristics, significant intergroup differences (P < 0.05) were identified in maximum nodule diameter, margin features, microcalcifications, and enhancement homogeneity. Furthermore, echogenicity, height-to-width ratio, enhancement intensity, and enhancement directionality showed more pronounced differences between groups (P < 0.01). However, no statistically significant differences were found in nodule location or intranodular vascularity (P > 0.05). Based on these findings, all characteristics demonstrating statistical significance were incorporated into subsequent model construction.

### 3.2 Construction of CEUS-based radiomics model

**Figure 7:**
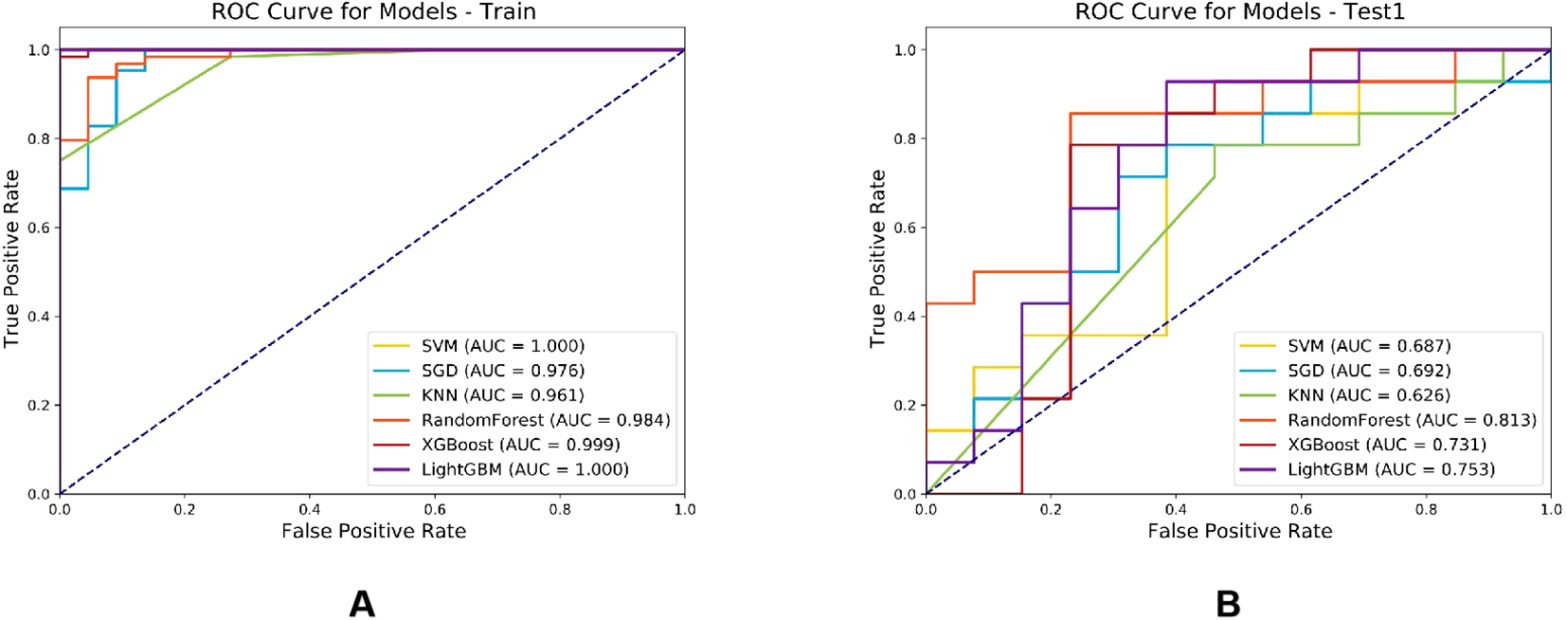
Comparison of ROC curves for six machine learning classifier models in the training cohort **(A)** and test cohort **(B)**.

Six machine learning classifiers including SVM, SGD, KNN, RF, XGBoost and LightGBM were constructed and their diagnostic performances were comprehensively evaluated. The AUC values in the training cohort were 1.000, 0.976, 0.961, 0.984, 0.999 and 1.000 respectively, while in the test cohort they were 0.687, 0.692, 0.626, 0.813, 0.731 and 0.753 respectively. Notably, the RF model demonstrated the highest diagnostic performance in the test cohort (AUC = 0.813) as shown in **Figure 7**. Furthermore, evaluation based on other metrics including accuracy, sensitivity and specificity also indicated superior performance of the RF model in the test cohort. Specifically, the RF model achieved an accuracy of 0.833, sensitivity of 0.875 and specificity of 0.769 in the test cohort, all of which were significantly higher than other models (**Table 2**). To further validate the robustness of the RF classifier, we performed leave-one-out cross-validation. Model stability assessed by leave-one-out cross-validation (LOO-CV) demonstrated tightly distributed AUC values (0.813, 95% CI: 0.797-0.864) with low coefficient of variation (CV=2.1%), confirming excellent reproducibility (**Supplementary Figure 1**, **Supplementary Table 2**).

**Table 2:** Diagnostic performance comparison of six machine learning classifiers.

Model	Cohort	Accuracy	Sensitivity	Specificity	AUC
SVM	Training cohort	1	1	1	1
	Test cohort	0.640	0.647	0.461	0.687
SGD	Training cohort	0.942	0.878	0.773	0.976
	Test cohort	0.640	0.651	0.630	0.692
KNN	Training cohort	0.919	0.856	0.727	0.961
	Test cohort	0.520	0.535	0.618	0.626
RF	Training cohort	0.988	0.977	0.954	0.984
	Test cohort	0.833	0.875	0.769	0.813
XGBoost	Training cohort	0.907	0.944	0.818	0.999
	Test cohort	0.760	0.761	0.714	0.731
LightGBM	Training cohort	1	1	1	1
	Test cohort	0.720	0.727	0.713	0.753

### 3.3 Construction and performance comparison of three radiomics models

**Figure 8:**
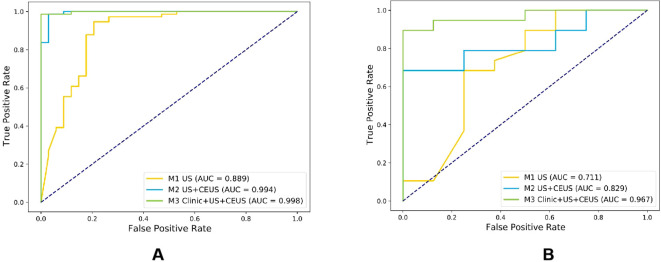
Comparison of ROC curves for three radiomics models in the training cohort **(A)** and testing cohort **(B)**.

Among the six CEUS radiomics models, the RF model demonstrated superior diagnostic performance. Therefore, we selected the RF model to further develop the US radiomics model (M1), US+CEUS radiomics model (M2), and clinical + US + CEUS radiomics model (M3), and systematically compared their diagnostic efficacy. As shown in **Figure 8**, ROC curve analysis revealed that the AUC values of the three models in the test set were M1 (0.889, 95% CI: 0.845–0.932), M2 (0.994, 95% CI: 0.949–1), and M3 (0.998, 95% CI: 0.955–1). In the independent validation cohort, the M3 model achieved significantly higher AUC (0.967, 95% CI: 0.898–1) compared to M1 (0.711, 95% CI: 0.641–0.780) and M2 (0.829, 95% CI: 0.758–0.900). Further quantitative analysis demonstrated that M3 exhibited superior performance metrics in the validation set, with accuracy (0.815), sensitivity (0.823), specificity (0.792), and F1-score (0.884) all exceeding those of M1 and M2 (**Table 3**).

**Table 3:** Comparison of diagnostic performance among three models.

Model	Cohort	Accuracy	Sensitivity	Specificity	F1-score
M1	Training cohort	0.879	0.920	0.867	0.917
	Test cohort	0.741	0.750	0.739	0.810
M2	Training cohort	0.926	1.000	0.902	0.949
	Test cohort	0.778	0.667	0.783	0.857
M3	Training cohort	0.917	0.947	0.892	0.943
	Test cohort	0.815	0.823	0.792	0.884

Although the M3 model demonstrated excellent overall performance, several misclassification cases were observed. For instance, one pathologically confirmed case of nodular goiter was misclassified as malignant by the model, exhibiting CEUS features of “heterogeneous enhancement” and “hypoenhancement,” which may overlap with the characteristics of malignant nodules in the training dataset. Another case of micro-papillary thyroid carcinoma (measuring <3 mm) was misclassified as benign, likely due to insufficient representation of small lesions in the training cohort.

### 3.4 Comparison of the effectiveness of radiomics models

**Figure 9:**
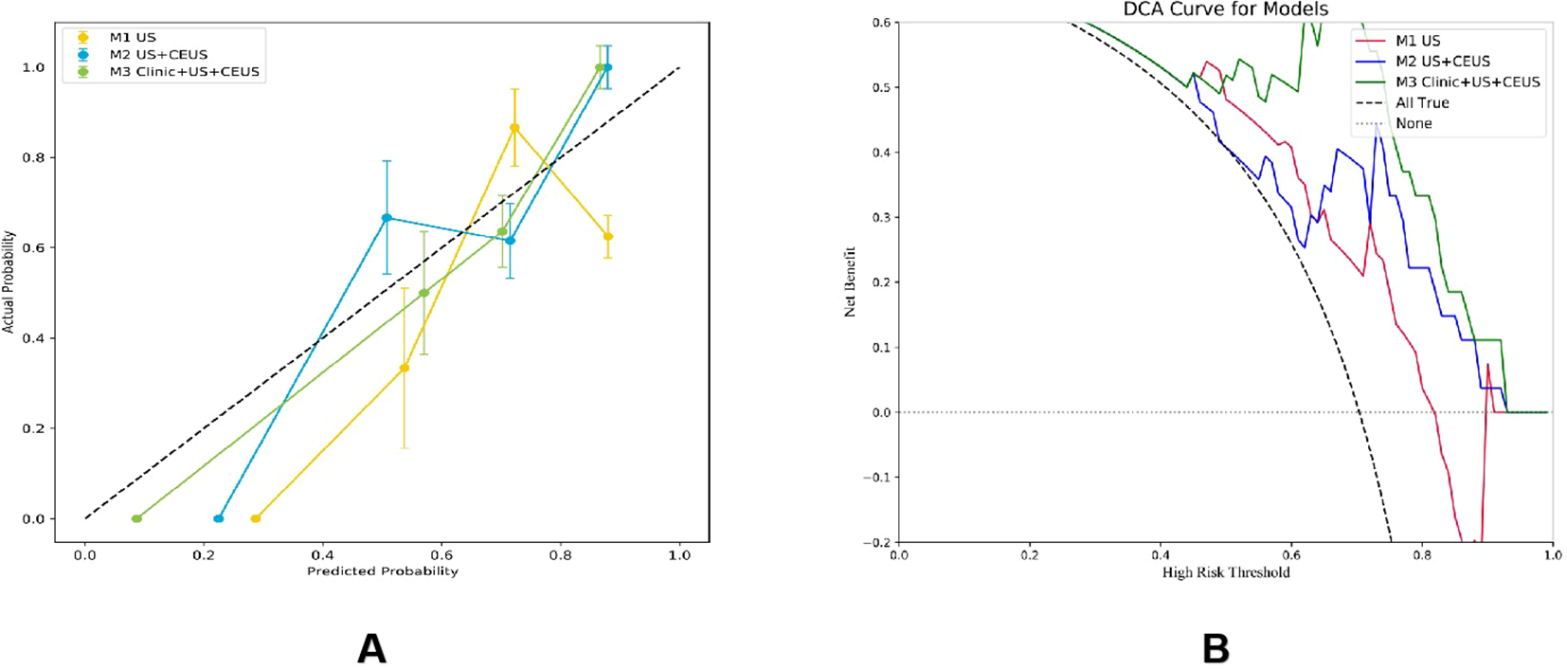
Calibration curves **(A)** and DCA **(B)** of three radiomics models.

The DeLong test was used to perform a statistical comparison of the area under the curve (AUC) for different radiomics models in diagnosing the benign and malignant thyroid nodules (TNs) in the test set. The detailed results are shown in **Table 4**. The analysis revealed that there were statistically significant differences in the AUC between the M1 model and the M2 and M3 models (P < 0.05), indicating that the diagnostic efficacy of the M2 and M3 models was significantly better than that of the M1 model. There was no statistical difference in the AUC between the M2 model and the M3 model (P > 0.05), suggesting that their diagnostic efficacies were comparable. Additionally, the calibration and DCA curves of M3 showed favorable consistency with reality (**Figure 9**).

**Table 4:** Comparison of AUC values for diagnostic efficacy of three models.

Model	AUC	*P-*value		
	(95% CI)	Comparison with M1	Comparison with M2	Comparison with M3
M1	0.711 (0.641, 0.780)	-	0.005*	0.011*
M2	0.829 (0.758, 0.900)	0.005*	-	0.078
M3	0.967 (0.898, 1)	0.011*	0.078	-

*P < 0.05 was considered to indicate a statistically significant difference.

## 4 Discussion

This study developed a multimodal radiomics model integrating CEUS radiomic features from three key timepoints, US parameters, and clinical data to accurately differentiate benign and malignant C-TIRADS 4 TNs. The results demonstrated that this multimodal model achieved outstanding diagnostic performance in the independent test cohort (AUC = 0.967), with accuracy, sensitivity, and specificity of 0.815, 0.823, and 0.792, respectively. Compared to single-modality models (US model AUC=0.711; US+CEUS model AUC=0.829), multimodal integration significantly improved diagnostic performance (Delong test P<0.05). This provides a robust decision-support tool to address overdiagnosis and overtreatment caused by the broad malignancy risk spectrum (2%-90%) of C-TIRADS 4 nodules.

The analysis of clinical and imaging characteristics of malignant nodules was generally consistent with previous studies (20–22). This study found that patients with malignant nodules were significantly younger than those with benign nodules (P<0.05), with conventional ultrasound features predominantly showing hypoechogenicity, taller-than-wide shape (height/width ratio >1), ill-defined margins, and microcalcifications. These imaging characteristics closely correlate with pathological changes in malignant tumors, including rapid cell proliferation, invasive growth, local necrosis, and calcification. Notably, although the mean diameter of malignant nodules was significantly smaller than benign nodules (9.25 ± 5.64 mm vs. 12.42 ± 12.16 mm, P<0.05), their vascular distribution patterns (CDFI) showed no statistically significant intergroup differences (P>0.05). This finding differs from some studies (20, 23).potentially due to; (1) larger benign nodules in our study being more detectable by CDFI due to higher vascular density, while newly formed microvessels in malignant nodules (with thinner diameters/slower flow) may fall below CDFI detection thresholds (24, 25); (2) technological advancements in ultrasound (e.g., superb microvascular imaging/SMI) improving microvascular detection capability (26), suggesting that reliance solely on the presence/absence of blood flow is no longer sufficient for effective nodule differentiation.

The advantage of CEUS technology lies not only in its ability to clearly display the microvascular structure within tissues and reflect the presence of blood vessels but also in its capacity for dynamic and quantitative analysis of nodule blood - perfusion conditions (such as enhancement patterns, time to peak, and post - peak changes and other related parameters) (11), These dynamic characteristics are the key to the differential diagnosis of benign and malignant nodules, thus providing richer imaging information for disease diagnosis. The innovative value of multi-timepoint CEUS analysis lies in overcoming the limitations of single-timepoint assessment by comprehensively capturing hemodynamic evolution.

This study innovatively selected three critical timepoints to construct the CEUS radiomics model: 2 seconds post-perfusion initiation (early perfusion), peak enhancement (peak phase), and 2 seconds post-peak (early washout). The scientific rationale is that: (1) The “2 seconds post-perfusion” phase reflects the initial contrast agent entry into the nodule, allowing preliminary evaluation of hemodynamic characteristics, particularly for identifying malignant tendencies through delayed perfusion; (2) The “peak enhancement” timepoint represents maximum contrast intensity within the nodule, reflecting peak blood perfusion. Malignant nodules typically demonstrate “heterogeneous hypoenhancement,” correlating with vascular heterogeneity and necrotic areas in malignant lesions (27). Analysis of enhancement intensity and distribution at peak provides crucial information about vascularization degree and perfusion homogeneity;(3) The “2 seconds post-peak” phase captures the washout process, where malignant nodules exhibit faster and more heterogeneous contrast washout, reflecting increased vascular permeability and unstable blood flow velocity. The CEUS radiomics model (RF algorithm) based on these three key timepoints achieved an AUC of 0.813 (sensitivity 0.875, specificity 0.769) in the test cohort, confirming the necessity of multi-timepoint analysis for comprehensive hemodynamic characterization. This aligns with the research direction of Chen JH et al. using multi-timepoint CEUS (19). However, while that study also employed multiple timepoints, our work innovatively selected three timepoints representing specific hemodynamic phases (early perfusion, peak, and early washout) rather than simply increasing quantity. Moreover, we were the first to deeply integrate these multi-timepoint CEUS features with conventional US characteristics and clinical data to construct a model, fully leveraging multimodal complementarity for synergistic diagnosis. In previous research, Li T et al. analyzed 302 CEUS cases with ring-enhanced nodules and found that ring-enhancement patterns could serve as important discriminators. Irregular hypo-ring enhancement was more frequent in malignant nodules, particularly those ≥10 mm, showing significantly higher specificity and AUC than C-TIRADS (92.8% vs 81.1%, P=0.021; 90.7% vs 82.3%, P=0.026) (28). While our study hasn’t yet explored different ring-enhancement patterns, future work could incorporate such specific patterns (e.g., irregular hypo-ring enhancement) into the feature system to further optimize the model.

The core advantage of multimodal integration lies in information complementarity and performance leap. Specifically, the CEUS radiomics model developed in this study demonstrated high diagnostic efficacy in differentiating C-TIRADS 4 TNs. The optimal random forest (RF) model achieved an accuracy of 0.833, sensitivity of 0.875, specificity of 0.769, and AUC of 0.813, indicating robust diagnostic performance in distinguishing benign from malignant thyroid nodules, consistent with previous studies (29, 30). Further incorporation of US imaging features showed comparable diagnostic performance between the CEUS radiomics model and the US+CEUS radiomics model (AUC: 0.813 vs. 0.829). The lack of significant improvement with the current sample size may be attributed to potential overlap or substitution of key US features by CEUS-derived characteristics, warranting further investigation with expanded cohorts to better assess the contribution of US features. The multimodal radiomics model significantly enhanced diagnostic efficacy, achieving an AUC of 0.967. This underscores that clinical data provide phenotype-specific information independent of imaging, thereby improving comprehensive diagnostic judgment and yielding superior performance with strong generalizability. The three data modalities—clinical parameters (phenotypic context), US (structural features), and CEUS (functional dynamics)—exhibit strong complementarity, collectively constructing a “multi-dimensional profile” of nodules. These results highlight the immense potential of multimodal fusion in addressing complex diagnostic challenges, demonstrating marked advancement over single-modality imaging (19, 31)or conventional radiomics models (29, 30).

The proposed RF model demonstrates potential for clinical implementation as a decision-support tool in thyroid nodule diagnosis. Integration into ultrasound workstations could provide radiologists with automated risk assessment for C-TIRADS category 4 nodules, potentially enhancing diagnostic confidence while reducing unnecessary biopsies. However, clinical adoption requires overcoming technical barriers (e.g., DICOM compatibility) and regulatory hurdles, which represent key challenges to be addressed in future studies.

The study has the following limitations: First, as a single-center retrospective study, it is subject to patient selection bias due to the higher malignancy risk of thyroid nodules in our referral center. Although stratified cross-validation and class weighting were applied for adjustment, synthetic oversampling techniques (e.g., SMOTE) were not used to preserve data authenticity. Future studies may consider incorporating resampling methods to further improve model performance. Second, the study lacks an external validation cohort, and future multi-center, multi-device prospective studies are needed to further verify the model’s generalizability. Finally, manual ROI delineation is time-consuming and labor-intensive; thus, automated segmentation algorithms should be developed to establish a standardized feature extraction pipeline.

## 5 Conclusion

Our study developed a high-performance multimodal diagnostic model through the innovative integration of radiomic features from three critical CEUS timepoints combined with conventional ultrasound and clinical data, establishing a novel decision-support tool for accurate noninvasive classification of C-TIRADS 4 thyroid nodules. The model’s superior diagnostic performance (AUC 0.967) demonstrates the transformative potential of multimodal integration in overcoming single-modality limitations and enhancing clinical decision-making, positioning this approach as a promising solution to mitigate unnecessary diagnostic procedures and overtreatment.

## Data Availability

The raw data supporting the conclusions of this article will be made available by the authors, without undue reservation.

## References

[1] Li YZ Teng D Ba JM Chen B Du JL He LJ. Efficacy and safety of long-term universal salt iodization on thyroid disorders: epidemiological evidence from 31 provinces of mainland China. Thyroid. (2020) 30:568–79. doi: 10.1089/thy.2019.0067, PMID: 32075540

[2] Kwak JY Han KH Yoon JH Moon HJ Son EJ Park SH. Thyroid imaging reporting and data system for US features of nodules: A step in establishing better stratification of cancer risk. Radiology. (2011) 260:892–9. doi: 10.1148/radiol.11110206, PMID: 21771959

[3] Tessler FN Middleton WD Grant EG Hoang JK Berland LL Teefey SA. ACR thyroid imaging, reporting and data system (TI-RADS): white paper of the ACR TI-RADS committee. J Am Coll Radiol. (2017) 14:587–95. doi: 10.1016/j.jacr.2017.01.046, PMID: 28372962

[4] Zhou J Yin L Wei X Zhang S Song Y Luo B. 2020 Chinese guidelines for ultrasound Malignancy risk stratification of thyroid nodules: the C-TIRADS. Endocrine. (2021) 70(2):256–79. doi: 10.1007/s12020-020-02441-y, PMID: 32827126

[5] Tessler FN Middleton WD Grant EG. Thyroid imaging reporting and data system (TI-RADS): A user’s guide. Radiology. (2018) 287:29–36. doi: 10.1148/radiol.2017171240, PMID: 29558300

[6] Cheng J Han B Chen YC Li Q Xia WW Wang NJ. Clinical risk factors and cancer risk of thyroid imaging reporting and data system category 4 A thyroid nodules. J Cancer Res Clin Oncol. (2024) 150(6):327. doi: 10.1007/s00432-024-05847-7, PMID: 38914743 PMC11196368

[7] Yin T Zheng B Lian Y Li H Tan L Xu S. Contrast-enhanced ultrasound improves the potency of fine-needle aspiration in thyroid nodules with high inadequate risk. BMC Med Imaging. (2022) 22:83. doi: 10.1186/s12880-022-00805-6, PMID: 35501723 PMC9063232

[8] Bartolotta TV Midiri M Galia M Runza G Attard M Savoia G. Qualitative and quantitative evaluation of solitary thyroid nodules with contrast-enhanced ultrasound: initial results. Eur Radiol. (2006) 16:2234–41. doi: 10.1007/s00330-006-0229-y, PMID: 16670868

[9] Cohen O Zhang J Zhang X Meng Y Chen Y. Contrast-enhanced ultrasound for the differential diagnosis of thyroid nodules: An updated meta-analysis with comprehensive heterogeneity analysis. PloS One. (2020) 15(4):e0231775. doi: 10.1371/journal.pone.0231775, PMID: 32310968 PMC7170259

[10] Liu Q Ouyang LQ Zhang SC Yang YX. Comparison of the value of ultrasound-guided fine needle aspiration biopsy and contrast-enhanced ultrasound in different sizes of thyroid nodules. Medicine. (2024) 103(39):e39843. doi: 10.1097/MD.0000000000039843, PMID: 39331869 PMC11441858

[11] Chen F Han H Wan P Chen L Kong W Liao H. Do as sonographers think: contrast-enhanced ultrasound for thyroid nodules diagnosis via microvascular infiltrative awareness. IEEE Trans Med Imaging. (2024) 43:3881–94. doi: 10.1109/TMI.2024.3405621, PMID: 38801692

[12] Guiot J Vaidyanathan A Deprez L Zerka F Danthine D Frix AN. A review in radiomics: Making personalized medicine a reality via routine imaging. Med Res Rev. (2022) 42:426–40. doi: 10.1002/med.21846, PMID: 34309893

[13] Cao LL Peng M Xie X Chen GQ Huang SY Wang JY. Artificial intelligence in liver ultrasound. World J Gastroenterol. (2022) 28:3398–409. doi: 10.3748/wjg.v28.i27.3398, PMID: 36158262 PMC9346461

[14] Gu JH Jiang TA. Ultrasound radiomics in personalized breast management: Current status and future prospects. Front Oncol. (2022) 12:963612. doi: 10.3389/fonc.2022.963612, PMID: 36059645 PMC9428828

[15] Zhou H Jin YH Dai L Zhang MW Qiu YQ Wang K. Differential diagnosis of benign and Malignant thyroid nodules using deep learning radiomics of thyroid ultrasound images. Eur J Radiol. (2020) 127:108992. doi: 10.1016/j.ejrad.2020.108992, PMID: 32339983

[16] Du H Chen F Li H Wang K Zhang J Meng J. Deep-learning radiomics based on ultrasound can objectively evaluate thyroid nodules and assist in improving the diagnostic level of ultrasound physicians. Quantit Imaging Med Surg. (2024) 14(8):5932–45. doi: 10.21037/qims-23-1597, PMID: 39144053 PMC11320491

[17] Guo SY Zhou P Zhang Y Jiang LQ Zhao YF. Exploring the value of radiomics features based on B-mode and contrast-enhanced ultrasound in discriminating the nature of thyroid nodules. Front Oncol. (2021) 11:738909. doi: 10.3389/fonc.2021.738909, PMID: 34722288 PMC8551634

[18] Ren J-Y Lv W-Z Wang L Zhang W Ma Y-Y Huang Y-Z. Dual-modal radiomics nomogram based on contrast-enhanced ultrasound to improve differential diagnostic accuracy and reduce unnecessary biopsy rate in ACR TI-RADS 4–5 thyroid nodules. Cancer Imaging. (2024) 24(1):17. doi: 10.1186/s40644-024-00661-3, PMID: 38263209 PMC10807093

[19] Chen J-H Zhang Y-Q Zhu T-T Zhang Q Zhao A-X Huang Y. Applying machine-learning models to differentiate benign and Malignant thyroid nodules classified as C-TIRADS 4 based on 2D-ultrasound combined with five contrast-enhanced ultrasound key frames. Front Endocrinol. (2024) 15:1299686. doi: 10.3389/fendo.2024.1299686, PMID: 38633756 PMC11021584

[20] Wettasinghe MC Rosairo S Ratnatunga N Wickramasinghe ND. Diagnostic accuracy of ultrasound characteristics in the identification of Malignant thyroid nodules. BMC Res Notes. (2019) 12:193–. doi: 10.1186/s13104-019-4235-y, PMID: 30940214 PMC6444851

[21] Rago T Vitti P. Risk stratification of thyroid nodules: from ultrasound features to TIRADS. Cancers. (2022) 14(3):717. doi: 10.3390/cancers14030717, PMID: 35158985 PMC8833686

[22] Moon HJ Kwak JY Kim MJ Son EJ Kim EK. Can vascularity at power doppler US help predict thyroid Malignancy? Radiology. (2010) 255:260–9. doi: 10.1148/radiol.09091284, PMID: 20308462

[23] Zhao D Jing Y Lin X Zhang B. The value of color Doppler ultrasound in the diagnosis of thyroid nodules: a systematic review and meta-analysis. Gland Surge. (2021) 10:3369–77. doi: 10.21037/gs-21-752, PMID: 35070897 PMC8749106

[24] Lyshchik A Moses R Barnes SL Higashi T Asato R Miga MI. Quantitative analysis of tumor vascularity in benign and Malignant solid thyroid nodules. J Ultrasound Med. (2007) 26:837–46. doi: 10.7863/jum.2007.26.6.837, PMID: 17526616

[25] Li F Huang F Liu C Pan D Tang X Wen Y. Parameters of dual-energy CT for the differential diagnosis of thyroid nodules and the indirect prediction of lymph node metastasis in thyroid carcinoma: a retrospective diagnostic study. Gland Surge. (2022) 11:913–26. doi: 10.21037/gs-22-262, PMID: 35694089 PMC9177276

[26] Luo H Yin L. Diagnostic value of superb microvascular imaging and color doppler for thyroid nodules: A meta-analysis. Front Oncol. (2023) 13:1029936. doi: 10.3389/fonc.2023.1029936, PMID: 37091165 PMC10113672

[27] Ma J-J Ding H Xu B-H Xu C Song L-J Huang B-J. Diagnostic performances of various gray-Scale, color doppler, and contrast-enhanced ultrasonography findings in predicting Malignant thyroid nodules. Thyroid. (2014) 24:355–63. doi: 10.1089/thy.2013.0150, PMID: 23978252

[28] Li T Mao L Wang X Li C Dong C Wu W. Ring-enhancement on CEUS: is it useful in the differential diagnosis of solid thyroid nodules? Ultrasonic Imaging. (2025) 47:37–44. doi: 10.1177/01617346241291511, PMID: 39428666

[29] Wang G Yin C Wang Y Li Q Yang D Wang P. Contrast-enhanced ultrasound (CEUS) characteristics of atypical-enhanced papillary thyroid carcinoma (PTC). Clin Hemorheol Microcirc. (2024) 88:71–9. doi: 10.3233/CH-242173, PMID: 38848170

[30] Zhou X Zhou P Hu Z Tian SM Zhao Y Liu W. Diagnostic efficiency of quantitative contrast-enhanced ultrasound indicators for discriminating benign from Malignant solid thyroid nodules. J Ultrasound Med. (2018) 37:425–37. doi: 10.1002/jum.14347, PMID: 28880412

[31] Huang Y Wang Y Liu L Zhu L Qiu Y Zuo D. VueBox^®^ perfusion analysis of dynamic contrast enhanced ultrasound provides added value in the diagnosis of small thyroid nodules. Clin Hemorheol Microcirc. (2023) 83:409–20. doi: 10.3233/CH-221681, PMID: 36683500

