# Supplementary material for: Multimodal radiomics model with triple -timepoint contrast-enhanced ultrasound for precise diagnosis of C-TIRADS 4 thyroid nodules
Source: Front Endocrinol (Lausanne). 2025 Aug 19;16:1639017. doi: 10.3389/fendo.2025.1639017 (PMC12401682; doi:10.3389/fendo.2025.1639017)
Supplement: Supplementary file 1 [file DataSheet1.pdf]

Supplementary Table 1 Comparison of Baseline Clinical Characteristics of Patients

Characteristics	Training cohort (n=108)	Test cohort (n=27)	*P*-value
Age (years)	50.14±10.51	47.59±10.24	0.260
Sex			
Male	86（79.63%）	17（62.96%）	
Female	22（20.37%）	10（37.04%）	0.069
Maximum nodule diameter (mm)	10.44±8.72	9.43±6.58	0.257
Location			
Isthmus	6（5.56%）	1（3.70%）	
Right lobe	61（56.48%）	13（48.15%）	
Left lobe	41（37.96%）	13（48.15%）	0.614
Echogenicity			
Markedly hypoechoic	6（5.55%）	2（7.41%）	
Hypoechoic	93（86.11%）	23（85.19%）	
Isoechoic	1（0.93%）	0（0.00%）	
Hyperechoic	1（0.93%）	1（3.70%）	
Mixed echogenicity	7（6.48%）	1（3.70%）	0.776
Shape (Aspect ratio)			
＜1	58（53.70%）	13（48.15%）	
＞1	50（46.30%）	14（51.85%）	0.605
Margin			
Regular	48（44.44%）	13（48.15%）	
Irregular	60（55.56%）	14（51.85%）	0.729
Microcalcification			
Yes	43（39.81%）	11（40.74%）	
No	65（60.19%）	16（59.26%）	0.930
Vascularity			
Yes	55（50.93%）	15（55.56%）	
No	53（49.07%）	12（44.44%）	0.667
Enhancement Intensity			
Hypoenhancement	67（62.04%）	18（66.67%）	
Isoenhancement	36（33.33%）	9（33.33%）	
Hyperenhancement	5（4.63%）	0（0.00%）	0.516

Continued

Characteristics	Training cohort (n=108)	Test cohort (n=27)	*P*-value
Enhancement Homogeneity			
Homogeneous	30（27.78%）	8（29.63%）	
Heterogeneous	78（72.22%）	19（70.37%）	0.848
Enhancement Directionality			
Centripetal	68（62.96%）	19（70.37%）	
Non-centripetal	40（37.04%）	8（29.63%）	0.472

*P < 0.05 was considered to indicate a statistically significant difference.

Supplementary Table 2 Robustness Analysis via Leave-One-Out Cross-Validation

Parameter	Value
Original AUC	0.813
LOO mean AUC ± SD	0.813 ± 0.017
LOO median AUC	0.810
Range (min–max)	0.798–0.864
Coefficient of variation (CV)	2.1%
Valid iterations	27/27

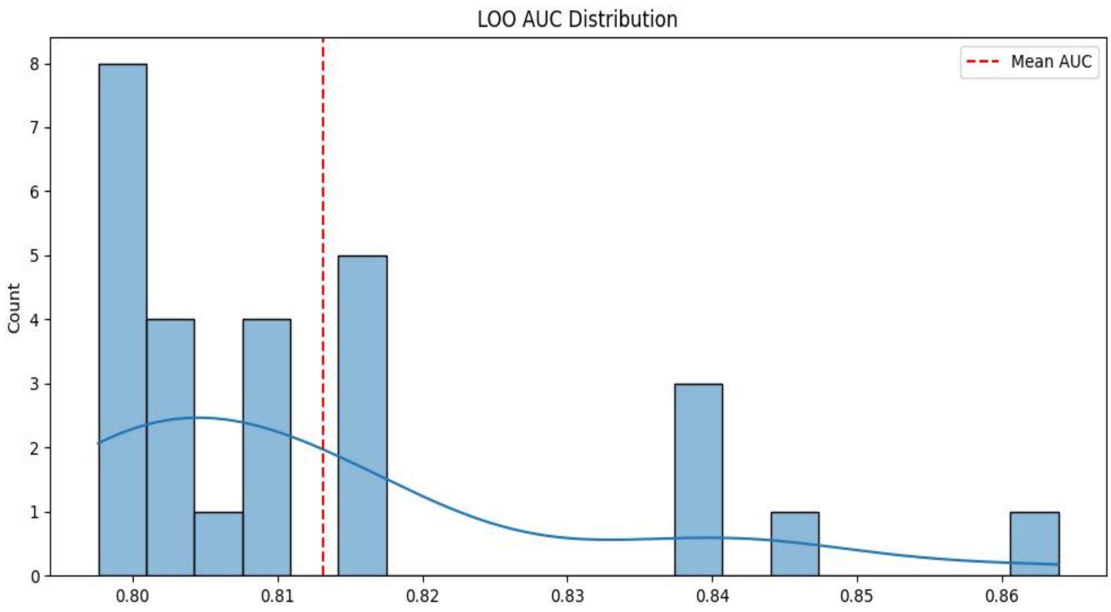

Supplementary Figure 1 Density distribution of AUC values from leave-one-out validation
